# A Dual-Laser Raman Strategy for Fast and Direct Detection and Quantification of Microplastics in Water

**DOI:** 10.3390/polym18091046

**Published:** 2026-04-25

**Authors:** Hongtaek Kim, Yong Ju Lee, Sangsig Kim

**Affiliations:** 1 Department of Micro Device Engineering, Korea University, Anam-ro 145, Seongbuk-gu, Seoul 02841, Republic of Korea; oriorioriori@korea.ac.kr; 2 Department of Forest Products and Biotechnology, Kookmin University, 77 Jeongneung-ro, Seongbuk-gu, Seoul 02707, Republic of Korea; paperlyj@kookmin.ac.kr; 3 School of Electrical Engineering, Korea University, Anam-ro 145, Seongbuk-gu, Seoul 02841, Republic of Korea

**Keywords:** backscattering, transmission, Raman spectroscopy, drinking water, real-time

## Abstract

Reliable quantification of microplastics in water remains challenging because most Raman-based methods require filtration, drying, or complex flow systems, which can lead to particle loss and signal instability. Here, we propose a simple dual-laser Raman strategy for the direct, real-time quantification of microplastics in water without pretreatment. By simultaneously integrating backscattering and transmission geometries using two identical 532 nm lasers, spatial variations in Raman scattering cross-sections, arising from particle motion and focal depth fluctuations, are effectively mitigated. The dual-laser configuration enhances Raman intensity by approximately 1.5-fold compared with backscattering and threefold compared with transmission alone (*p* < 0.001), enabling robust real-time detection with a temporal resolution of 0.1 s. Accurate particle counting is demonstrated using polystyrene (PS) standard beads and further validated for polyamide 6 (PA6) and polyvinyl chloride (PVC) particles with irregular morphologies and broad size distributions, with no false-positive events observed. By prioritizing simplicity and quantitative reliability over ultimate size resolution, the proposed strategy provides a practical approach for routine monitoring of microplastics in drinking water and industrial aqueous systems.

## 1. Introduction

Microplastics are commonly defined as plastic particles ranging in size from approximately 1 µm to 5 mm, originating from plastic products manufactured and used in human activities [1,2,3]. Based on their origin, microplastics are generally classified as either primary microplastics, which are intentionally produced at small sizes, or secondary microplastics, which result from the fragmentation and weathering of larger plastic materials under environmental stressors such as ultraviolet radiation, mechanical abrasion, climatic conditions, and biological activity [1,4].

Recent studies have reported the widespread presence of microplastics in drinking water, including both tap and bottled sources, raising increasing concerns regarding human exposure through ingestion and inhalation [4,5]. The potential health impacts of microplastics depend on several factors, including particle size, polymer composition, and the presence of hazardous chemicals adsorbed onto particle surfaces. Common polymers identified in drinking water include polypropylene (PP), polystyrene (PS), polyethylene (PE), polyethylene terephthalate (PET), polyamide (PA), polyurethane (PU), and polyvinyl chloride (PVC), with particle sizes spanning from the submicrometer to millimeter scale [6,7,8]. Consequently, there is a growing demand for analytical methods that enable not only chemical identification but also reliable quantitative determination of microplastic particle concentrations in water.

**Figure 1:**
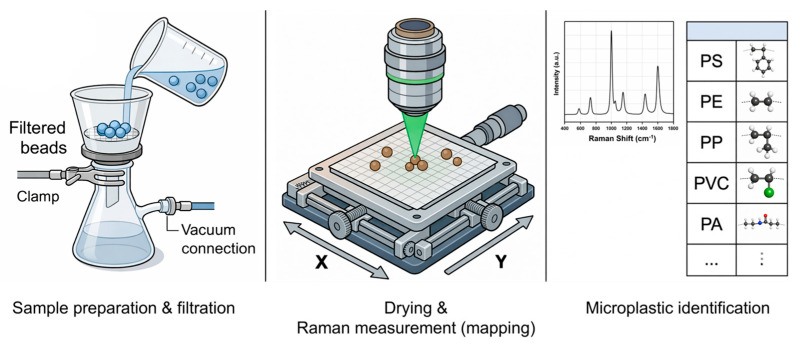
Conventional filtration-based Raman measurement methods for microplastic detection.

Various analytical techniques have been employed for microplastic analysis, including Fourier transform infrared spectroscopy (FT-IR), Raman spectroscopy, and gas chromatography–mass spectrometry (GC–MS). Among these, Raman spectroscopy offers a distinct advantage in spatial resolution and chemical identification [7,9,10,11]. When combined with optical microscopy, it can achieve resolutions on the order of ~1 µm, enabling the analysis of particles smaller than those typically accessible by conventional FT-IR methods, which are generally limited to sizes of approximately 10 µm [12,13]. Despite this advantage, most Raman-based analyses of microplastics rely on filtration and drying prior to measurement [14,15]. Such pretreatment steps frequently result in particle loss, aggregation, and morphological deformation, thereby limiting the reliability of quantitative analyses, particularly accurate particle counting [14,15,16,17,18]. Moreover, conventional filtration-based approaches (Figure 1) restrict the direct analysis of untreated water samples and often require disposal of the original sample, reducing overall analytical efficiency and reproducibility.

To address these limitations, recent studies have explored the direct Raman detection of microplastics suspended in water, including flow-based Raman systems designed for real-time analysis of moving particles [19,20,21]. In such dynamic systems in particular, establishing a rigorous limit of detection (LOD) remains a critical challenge due to the inherent trade-off between particle velocity and the signal integration time required for distinct spectral identification [22]. Although these approaches have demonstrated high sensitivity and, in some cases, the ability to detect particles down to a few micrometers, they typically require complex system architectures, such as precise flow control, particle focusing mechanisms, acoustic components, or polymer-specific measurement conditions [20,21,23]. As a result, despite their technical sophistication, their practical implementation for routine drinking water analysis and industrial monitoring remains challenging [24,25,26].

In many environmental and regulatory contexts, the importance of robust and reproducible quantification of microplastic particle numbers is increasingly recognized as more critical than achieving the smallest possible particle size resolution [27,28]. Accordingly, there is a clear need for a simple Raman-based analytical strategy that enables real-time quantification of microplastics directly in water without extensive pesretreatment or system complexity.

In this study, we propose a dual-laser Raman strategy that combines backscattering and transmission geometries to improve signal robustness during real-time measurements. By simultaneously illuminating the same measurement volume from opposite directions, the approach mitigates signal fluctuations caused by particle motion and focal instability. Rather than prioritizing ultimate size resolution, the proposed method emphasizes simplicity, robustness, and quantitative reliability. The performance of this strategy is demonstrated using representative polymers, including PS, PA6, and PVC, highlighting its applicability to structurally and compositionally diverse microplastics relevant to drinking water and industrial environments.

## 2. Materials and Methods

### 2.1. Materials

PS microspheres with a nominal diameter of 100 µm were purchased as certified standard beads from Thermo Fisher Scientific (Fremont, CA, USA) and used to validate the quantification accuracy under controlled conditions. PA6 and PVC particles were obtained from commercially available polymer products to represent microplastics encountered in realistic environmental and industrial contexts. All samples were handled exclusively with glassware to minimize plastic contamination. A stainless steel mesh filter (30 µm nominal pore size; 30 × 60 µm aperture, Anping Pinshang Wire Mesh Products Co., Ltd, Hengshui, China) was used for particle recovery and verification.

### 2.2. Quality Assurance and Quality Control (QA/QC)

To ensure analytical reliability and prevent contamination, all experiments were conducted using exclusively glassware pre-cleaned with ultrapure water (Milli-Q, Merck Millipore, Burlington, MA, USA; 18.2 MΩ·cm at 25 °C). Blank samples, consisting of the same ultrapure water used for particle suspensions, were routinely measured within the glass capillary prior to each experiment. The absence of Raman peaks in these blank spectra confirmed that the water and the capillary were free from background microplastic contamination. Furthermore, all sample handling was performed in a controlled environment to minimize airborne fiber interference.

### 2.3. Sample Preparation

**Figure 2:**
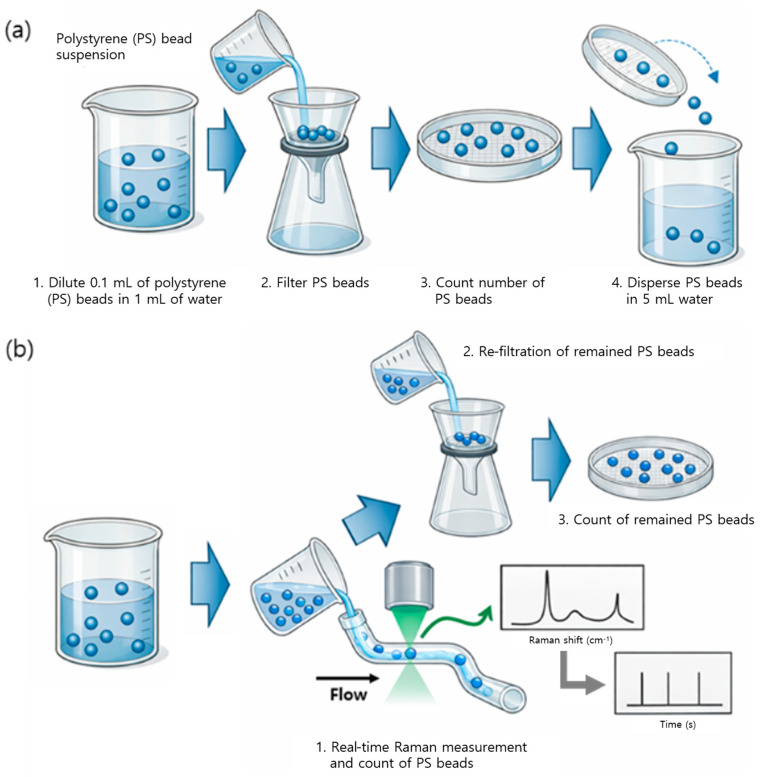
Conceptual illustration of Raman-based microplastic analysis methods: (**a**) a conventional workflow relying on filtration and offline Raman measurement, and (**b**) the proposed direct Raman strategy for real-time detection and quantification of microplastics in water.

For the PS samples, a known number of standard beads was manually transferred into a glass container filled with ultrapure water. The particle count was visually confirmed prior to Raman measurements (Figure 2a).

PA6 and PVC samples were mechanically fragmented from bulk materials and subsequently dispersed in water. Their particle size distributions were evaluated using optical microscopy and a laser diffraction particle size analyzer to confirm that the particles were within the micrometer size range relevant to this study.

No filtration, drying, or chemical pretreatment was applied to any sample. All Raman measurements were conducted directly on microplastic particles suspended in water to preserve their native aqueous state.

### 2.4. Optical Microscopy and Particle Size Characterization

Optical microscopy images of PS, PA6, and PVC particles were acquired using a digital microscope (BXFM, Olympus, Tokyo, Japan) equipped with a calibrated measurement scale. Representative images were used to evaluate particle morphology and estimate particle size.

The particle size distributions of PA6 and PVC were further characterized using a laser diffraction particle size analyzer (LA-960 V2, HORIBA, Kyoto, Japan). The measured distributions confirmed that the majority of particles were within the micrometer to submillimeter size range.

### 2.5. Raman Spectroscopy Setup and Measurement Conditions

**Figure 3:**
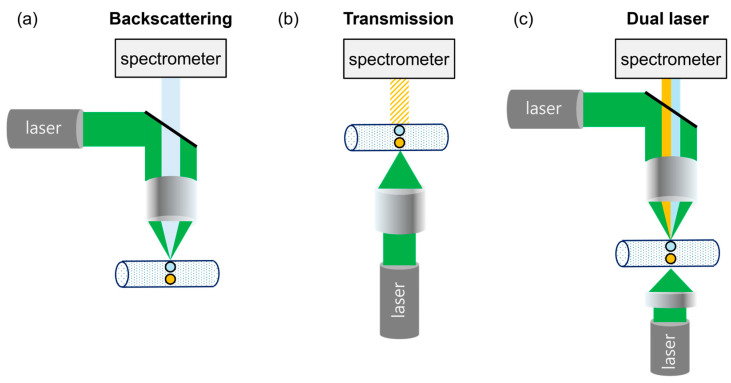
Schematic illustration of Raman excitation geometries used in this study: (**a**) conventional backscattering geometry, (**b**) conventional transmission geometry, and (**c**) the proposed dual-laser configuration combining both geometries to illuminate the same measurement volume from opposite directions. In the schematics, the green paths represent the 532 nm excitation laser beams. The light blue paths indicate the backscattering Raman signals from the light blue particles, while the yellow paths represent the transmission Raman signals from the yellow particles. The cylindrical tube depicts the water-filled glass capillary.

Raman measurements were performed using a custom-built dual-laser system based on a commercial micro-Raman spectrometer (LabRAM Soleil, HORIBA, Loos, France) coupled with a Nikon microscope (Nikon, Tokyo, Japan). To implement the simultaneous dual-excitation strategy, two identical 532 nm lasers maintained at 70 mW were integrated into the setup. As illustrated in Figure 3, for backscattering geometry, a Nikon x5 objective lens (TU Plan Fluor, Nikon, Tokyo, Japan) was used, while a macro lens was utilized for the transmission path. The measurement volume was localized within a glass capillary (inner diameter: 380 µm, outer diameter: 430 µm), fabricated in-house using disposable glass Pasteur pipettes (Marienfeld Superior, Lauda-Königshofen, Germany), which was mounted directly on the stage of the Raman system. All time-resolved spectra were acquired with an integration time of 0.1 s.

In conventional Raman spectroscopy, either backscattering or transmission geometry is typically employed. In the backscattering configuration, the Raman signal intensity is highly sensitive to focal alignment between the excitation beam and the particle; small deviations from the focal plane can result in significant reductions in signal intensity [29]. In contrast, the transmission geometry is less sensitive to focal position because the excitation beam traverses the entire sample thickness [30]; however, the collected Raman signal is generally weaker due to reduced collection efficiency.

To address these complementary limitations, a dual-laser Raman configuration was implemented by combining backscattering and transmission geometries (Figure 3). The two excitation beams were aligned to illuminate the same measurement volume from opposite directions, enabling simultaneous Raman excitation under both conditions. This configuration reduces position-dependent signal fluctuations associated with particle motion by providing complementary excitation and collection pathways.

The dual-laser system operated without flow control, particle-focusing mechanisms, or complex optical alignment. All measurements were conducted under static or free-moving particle conditions in water (Figure 2b), enabling reproducible Raman signal acquisition with short integration times suitable for real-time analysis.

Raman-scattered light was collected using a microscope objective lens and directed to a spectrometer equipped with a thermoelectrically cooled charge-coupled device (CCD) detector. Spectra were recorded over the range of 200–3500 cm^−1^ with a spectral resolution of approximately 4 cm^−1^.

### 2.6. Raman Measurement Conditions

All Raman spectra were acquired with an integration time of 0.1 s per spectrum to enable real-time measurements. The laser power was maintained below levels that could induce thermal damage, bubble formation, or other perturbations in the aqueous medium.

Time-resolved Raman spectra were continuously recorded while microplastic particles moved freely in water. Polymer identification was based on characteristic Raman bands specific to each material: 1002 cm^−1^ for PS, 1638 cm^−1^ for PA6, and 628 cm^−1^ for PVC.

### 2.7. Data Processing and Quantification Criteria

Raman spectra were processed with baseline correction to remove background fluorescence using LabSpec 6 software (HORIBA, Loos, France). To mitigate spectral variability from water, characteristic polymer peaks (1002 cm^−1^ for PS, 1638 cm^−1^ for PA6, and 628 cm^−1^ for PVC) were specifically monitored as they exhibit minimal overlap with the broad Raman bands of water. Furthermore, it should be noted that because this strategy relies on a single-particle counting paradigm rather than bulk mass-concentration measurements, traditional limits of detection (LOD) and quantitation (LOQ) are conceptualized differently. In this dynamic system, the LOD is defined by the minimum detectable particle size that yields a Raman signal exceeding the preset signal-to-noise (S/N) threshold. Conversely, the upper LOQ is inherently restricted by the temporal resolution (0.1 s) and the probability of coincidence errors when multiple particles pass through the focal volume simultaneously. A particle detection event was defined as the appearance of a characteristic, polymer-specific Raman peak exceeding a predefined signal-to-noise threshold.

Real-time particle counting was performed by identifying discrete Raman detection events as a function of time. No post-measurement filtering, temporal averaging, or manual correction was applied, ensuring that the reported particle counts directly reflect the raw measurement output.

## 3. Results and Discussion

### 3.1. Comparison of Raman Efficiency Between Measurement Geometries

**Figure 4:**
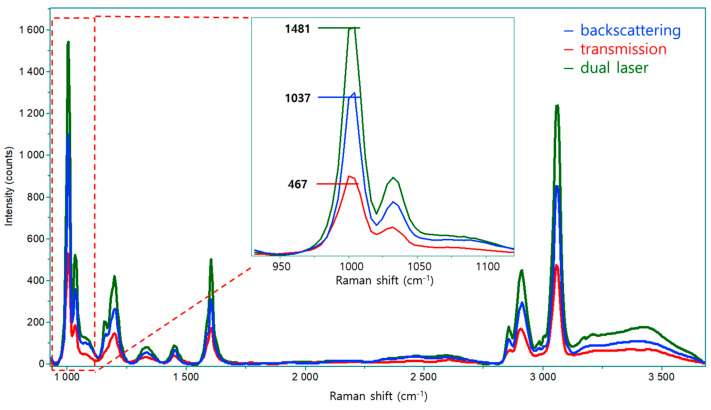
Comparison of Raman signal intensities for polystyrene (PS) beads under backscattering, transmission, and dual-laser configurations.

Direct Raman detection of microplastics suspended in water is inherently challenged by particle motion and focal instability during measurement [7]. Unlike solid samples fixed on a substrate, microplastic particles in an aqueous environment continuously move through the laser focal volume, resulting in significant fluctuations in Raman signal intensity [21]. This instability becomes particularly problematic when short acquisition times are required for real-time analysis and particle counting [31]. To address this limitation, a dual-laser Raman configuration was employed (Figure 3c). As an initial step in evaluating this system, the Raman efficiencies associated with different measurement geometries were systematically examined (Figure 4).

To quantitatively assess the effectiveness of the dual-laser strategy, Raman signal intensities obtained under backscattering, transmission, and dual-laser configurations were compared using three independent measurements (n = 3) for each setup (Figure S1, Supplementary Information). Polystyrene (PS) beads were selected as representative particles due to their well-defined Raman signature, particularly the strong aromatic ring breathing mode at 1002 cm^−1^ [32,33]. As shown in Figure 4, the backscattering configuration produced an average Raman intensity of approximately 1036.7 ± 9.1 counts, whereas the transmission configuration yielded a significantly lower average intensity of approximately 467.0 ± 3.5 counts under identical acquisition conditions. When both geometries were applied simultaneously, the dual-laser configuration generated an average intensity of approximately 1481.6 ± 8.1 counts. This corresponds to a statistically significant enhancement factor of approximately 1.5 relative to backscattering alone (*p* < 0.001, Student’s *t*-test) and approximately threefold relative to transmission alone (*p* < 0.001, Student’s *t*-test).

In addition to increased signal intensity, the dual-laser configuration exhibited improved signal stability during time-resolved measurements. The abrupt intensity drops frequently observed in single-geometry measurements were substantially reduced, enabling consistent detection with short integration times of 0.1 s. These results demonstrate that the dual-laser approach provides a practical means of improving both Raman efficiency and signal robustness without increasing system complexity.

### 3.2. Raman Spectral Characteristics of Representative Polymers Under Dual-Laser Excitation

**Figure 5:**
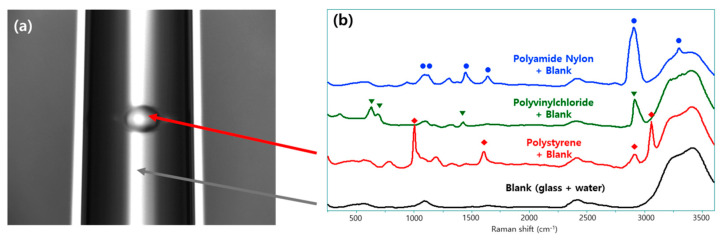
Raman analysis of microplastics under dual-laser excitation: (**a**) optical image showing the measurement configuration, in which a single polymer particle suspended in water is positioned within a glass capillary (indicated by the arrow); (**b**) representative Raman spectra of polyamide 6 (PA6), polyvinyl chloride (PVC), and polystyrene (PS) particles measured in water, together with the corresponding blank spectrum obtained from a water-filled glass capillary. Note: The red arrows conceptually illustrate that each spectrum was acquired from an individual particle localized within the capillary. The spectra represent distinct, separate measurements for each polymer type, not a simultaneous acquisition from a single particle.

Following the observed improvements in Raman efficiency and signal stability achieved with the dual-laser configuration (Figure 4), the applicability of this approach to different polymer types was evaluated. Raman spectra of PS, PA6, and PVC were analyzed under dual-laser excitation. Representative spectra for each polymer are shown in Figure 5, and the corresponding band assignments are summarized in Table 1.

PS particles exhibited a prominent Raman band at 1002 cm^−1^, corresponding to the aromatic ring breathing mode, along with additional characteristic peaks at 620 and 1602 cm^−1^. These features provide a strong and unambiguous spectral fingerprint for PS identification and serve as reliable markers for real-time particle detection.

PA6 particles displayed characteristic amide-related Raman bands, including the amide I band at approximately 1638 cm^−1^ and CH_2_ stretching vibrations near 2930 cm^−1^. In contrast, PVC particles exhibited distinct Raman features associated with C–Cl stretching and CH_2_ deformation modes, with prominent peaks observed at approximately 628 and 1427 cm^−1^.

The distinct and non-overlapping Raman fingerprints of PS, PA6, and PVC enabled reliable polymer identification without modification of measurement conditions or laser parameters. These results confirm that the dual-laser strategy can be broadly applied to chemically diverse microplastics using polymer-specific Raman markers for real-time detection and quantification.

### 3.3. Sample Morphology and Size Distribution in Relation to Quantification Robustness

In real-world environments, such as drinking water and industrial systems, microplastics predominantly exist as irregularly shaped particles with broad size distributions due to continuous fragmentation and weathering, in contrast to idealized spherical standard beads [1]. Accordingly, the effects of particle morphology and size distribution on Raman detection performance were investigated. The robustness of the dual-laser strategy against particle heterogeneity is attributed to an increased effective detection volume and reduced sensitivity to focal instability [38].

**Figure 6:**
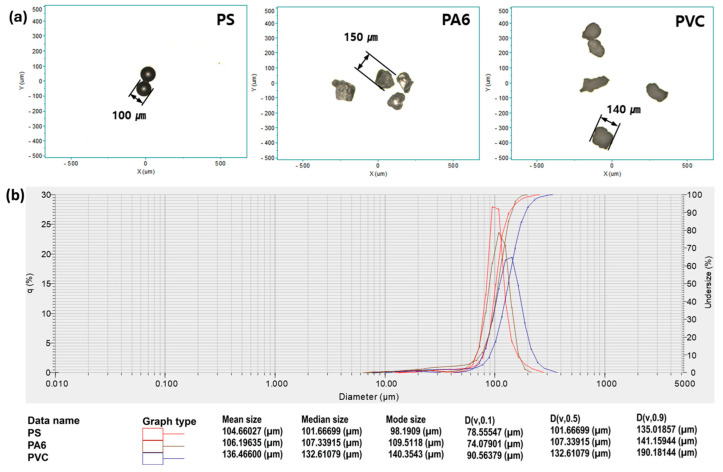
Optical images and particle size distributions of polystyrene (PS), polyamide 6 (PA6), and polyvinyl chloride (PVC) microplastics: (**a**) representative optical microscopy images showing particle morphology and characteristic sizes; (**b**) diameter-based particle size distributions expressed as volume-based frequency (q), together with the corresponding characteristic diameters.

Optical microscopy images (Figure 6a) show that PS standard beads exhibit uniform spherical shapes with narrow size distributions, whereas PA6 and PVC particles derived from commercial polymers display irregular morphologies and broader size distributions. Laser diffraction measurements (Figure 6b) further confirm that PA6 and PVC particles span a wide size range, from the micrometer to the submillimeter scale, representative of heterogeneous microplastics commonly encountered in practical environments.

Despite these differences in particle shape and size distribution, stable real-time Raman detection and quantification were achieved for all polymer types using the dual-laser configuration, as shown in Figure 5. These results indicate that variations in particle morphology and size distribution do not significantly affect the stability of Raman signal acquisition under the dual-laser configuration.

### 3.4. Real-Time Detection and Quantification of Polymer Particles

As an initial demonstration and mechanistic proof-of-concept, the feasibility of real-time microplastic quantification using the proposed dual-laser Raman strategy was evaluated with PS beads. Six PS beads were dispersed in water, and Raman spectra were continuously acquired with an integration time of 0.1 s over a total duration of 1000 s.

**Figure 7:**
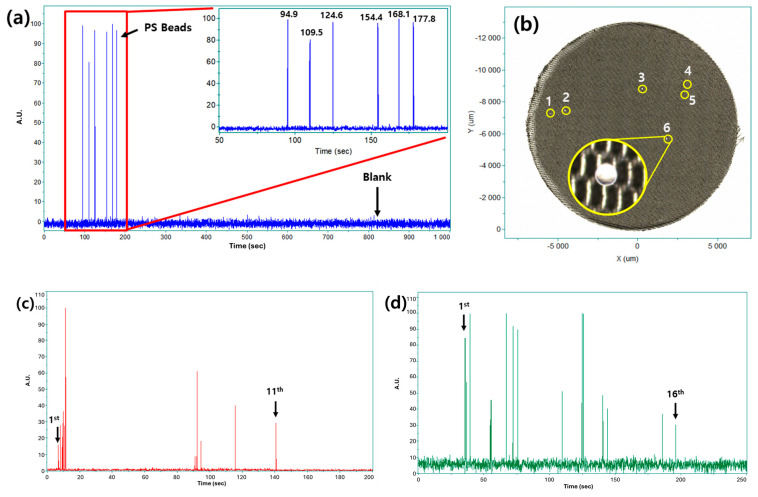
Real-time Raman quantification of microplastic particles in water: (**a**) detection of six polystyrene (PS) beads; (**b**) optical microscopy image of the prepared PS beads, where the numbers indicate the total count of the filtered beads; (**c**) detection of eleven polyamide 6 (PA6) particles; and (**d**) detection and counting of sixteen polyvinyl chloride (PVC) particles.

As shown in Figure 7a, six distinct Raman events corresponding to the characteristic PS band at 1002 cm^−1^ were detected at 94.9, 109.5, 124.6, 154.4, 168.1, and 177.8 s. Each event was clearly resolved in time, demonstrating that individual microplastic particles can be sequentially detected as they pass through the measurement volume.

To independently verify quantification accuracy, the measured PS suspension was subsequently filtered using a stainless steel mesh filter (30 μm pore size). As shown in Figure 7b (and further detailed in Figure S2, Supplementary Information), six PS beads were observed on the filter, in complete agreement with the number of Raman-detection events. While transparent PS beads inherently exhibit low visual contrast against common mesh filters in brightfield microscopy, careful manual observation confirmed the successful recovery of exactly six particles. This direct physical count establishes a robust ground truth for quantitative accuracy without introducing the procedural loss errors associated with traditional ex situ cross-validation methods. This result confirms that the proposed method enables accurate particle counting under low-particle-number conditions without false-positive detection. Although data acquisition extended beyond 1000 s, all six Raman events were detected within approximately 200 s. This observation highlights the rapid nature of the approach and indicates that reliable quantification of small numbers of microplastics can be achieved on timescales significantly shorter than those required for conventional filtration-based methods.

To further evaluate applicability beyond PS, real-time Raman quantification experiments were conducted using PA6 and PVC microplastic particles. Suspensions containing 11 PA6 particles and 16 PVC particles were prepared following the same procedure used for PS. The detected Raman events are summarized in Figure 7c,d.

Distinct Raman events corresponding to the characteristic polymer-specific bands of PA6 and PVC were detected in real time for both samples. The number of detected events matched the prepared particle counts, indicating that the dual-laser strategy enables accurate quantification across chemically distinct microplastics. The PA6 and PVC particles, obtained from commercially available polymer products, exhibited irregular morphologies and broader size distributions compared to the PS standard beads. Despite this heterogeneity, stable Raman detection and accurate quantification were achieved without modifying measurement conditions or laser configuration.

These results demonstrate that the proposed dual-laser Raman strategy is not limited to idealized standard materials but is also applicable to realistic microplastic samples. The ability to simultaneously identify polymer type and quantify particle number in water highlights the potential of this approach for practical microplastic analysis in environmental and industrial contexts.

### 3.5. Quantification Performance Under Increased Particle Numbers

**Figure 8:**
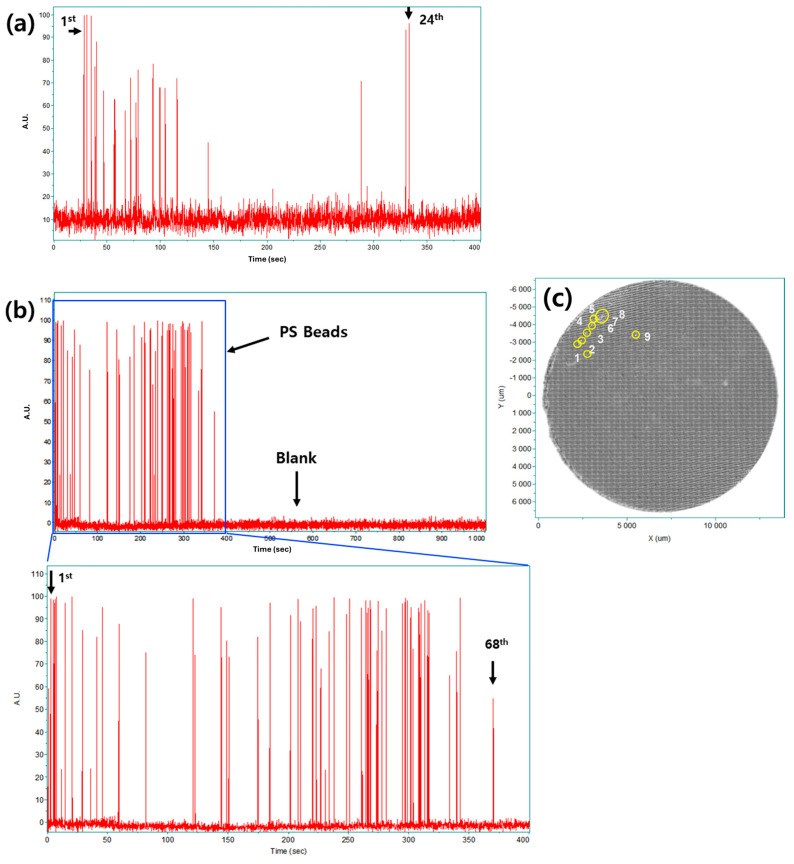
Additional real-time Raman quantification results: (**a**) detection of twenty-four polystyrene (PS) beads; (**b**) detection of sixty-eight PS beads from an initial suspension of seventy-seven beads; and (**c**) optical microscopy image of the nine undetected PS beads (indicated by the numbers 1–9) recovered from the sample container, confirming incomplete particle transfer.

To rigorously validate the true quantitative robustness of the proposed method, Raman measurements were performed on PS suspensions containing larger particle populations. Dispersions containing 24 and 77 PS beads were prepared, and the detected Raman events are summarized in Figure 8.

For the sample containing 24 PS beads, all particles were successfully detected using dual-laser Raman measurements, resulting in a quantification accuracy of 100% (Figure 8a). This demonstrates that the proposed strategy maintains quantitative reliability beyond low-particle-number conditions.

In contrast, 68 Raman events were detected in the suspension containing 77 PS beads (Figure 8b). To investigate the discrepancy, the original sample container was re-filtered, and exactly 9 beads were recovered from the beaker (Figure 8c). This confirms that the missing counts were not due to detection failure but rather to incomplete physical transfer (pipetting/pumping loss) of particles into the capillary volume. By physically accounting for the exact number of lost particles, this physical mass-balance approach verifies a near-complete detection accuracy and complete analytical recovery for all particles that successfully passed through the focal zone.

No false-positive Raman events were observed in any measurement. These results demonstrate that the proposed dual-laser strategy enables reliable particle counting, while deviations observed at higher particle numbers are primarily attributable to sample handling rather than to intrinsic limitations of the analytical method.

## 4. Conclusions

This study presents a dual-laser Raman strategy for the direct, real-time quantification of microplastics in water without the need for filtration, drying, or flow control. By integrating backscattering and transmission geometries, the approach reduces signal instability arising from particle motion and focal sensitivity, thereby enabling stable detection with short acquisition times. Accurate real-time quantification was demonstrated for polystyrene standard beads, as well as for polyamide 6 and polyvinyl chloride particles exhibiting irregular morphologies and broad size distributions, without false-positive detection. While this proof-of-concept establishes a robust optical foundation in ultrapure water, the system is designed with scalability in mind. Future integration of advanced baseline correction and spectral deconvolution algorithms will further enhance its performance in complex matrices (e.g., wastewater or high turbidity) and expand its applicability to highly heterogeneous realistic samples, including microfibers and complex polymer blends. By prioritizing simplicity and quantitative reliability over ultimate size resolution, the proposed strategy provides a practical and transformative approach for routine monitoring of microplastics in drinking water and industrial aqueous systems.

## Figures and Tables

**Table 1:** Representative Raman peak positions and assignments for polystyrene (PS), polyamide 6 (PA6), and polyvinyl chloride (PVC).

Polymers	Peak Positions, cm^−1^	Assignment	References
Polystyrene	621	Ring deformation mode	[32,33]
	792	C-H out-of-plane deformation	
	1002	Ring breathing mode	
	1032	C-H in-plane deformation	
	1191	C-C stretching	
	1449	CH_2_ scissoring	
	1601	C=C stretch	
	2854, 2909	C_sp3_-H stretching	
	3058	C_sp2_-H stretching	
Polyamide 6	932	C-C stretching	[34,35]
	1128	C-N stretching	
	1280	Amide III	
	1446, 1469	CH_2_ deformation	
	1638	Amide I	
	2874, 2902, 2928	C_sp3_-H stretching	
	3300	N-H stretching	
Polyvinylchloride	628	C-Cl stretching	[36,37]
	692	C-Cl deformation	
	1093, 1178	C-C stretching	
	1249	CH wagging + C-Cl coupling	
	1427	CH_2_ deformation	
	2847, 2911, 2937, 2971	C_sp3_-H stretching	

## Data Availability

The original contributions presented in this study are included in the article/Supplementary Materials. Further inquiries can be directed to the corresponding author.

## Supplementary Materials

The following supporting information can be downloaded at: https://www.mdpi.com/article/10.3390/polym18091046/s1, Figure S1. Comparison of Raman signal intensities for a single PS bead across different excitation geometries; Figure S2. Optical microscope image of the successfully recovered six PS beads on a mesh filter; Figure S3. Visual validation of the signal-to-noise (S/N) threshold for real-time particle detection; Table S1. Raw data of Raman intensities (counts) and statistical analysis (Student’s two-tailed *t*-test) evaluating the signal enhancement of the dual-laser configuration compared to conventional single-geometry setups.
