# Supplementary material for: A Dual-Laser Raman Strategy for Fast and Direct Detection and Quantification of Microplastics in Water
Source: Polymers (Basel). 2026 Apr 25;18(9):1046. doi: 10.3390/polym18091046 (PMC13165335; doi:10.3390/polym18091046)
Supplement: Supplementary file 1 [file polymers-18-01046-s001.zip › polymers-4250968-supplementary.pdf]

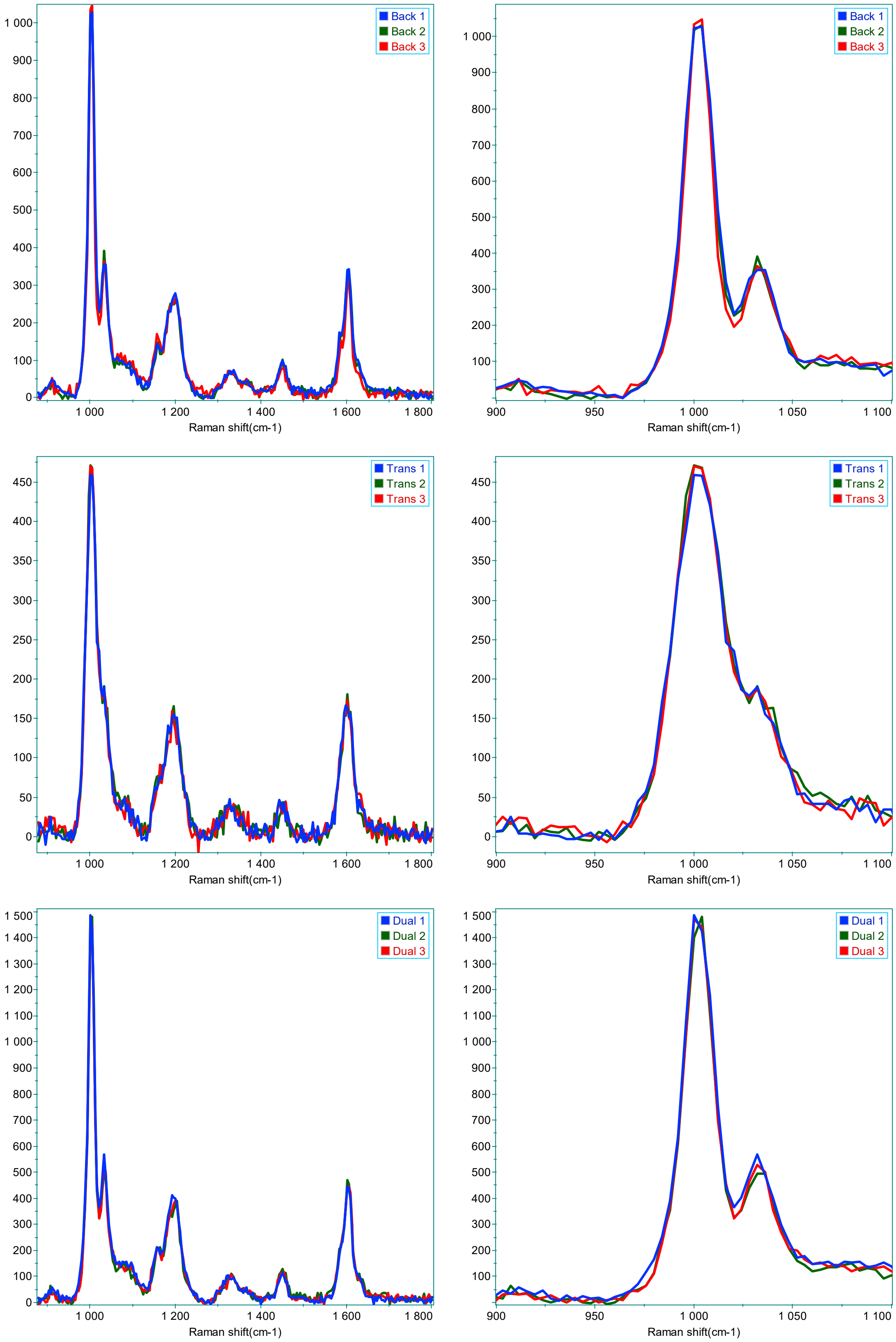
Back 1
Back 2
Back 3
Trans 1
Trans 2
Trans 3
Dual 1
Dual 2
Dual 3
Raman shift(cm-1)

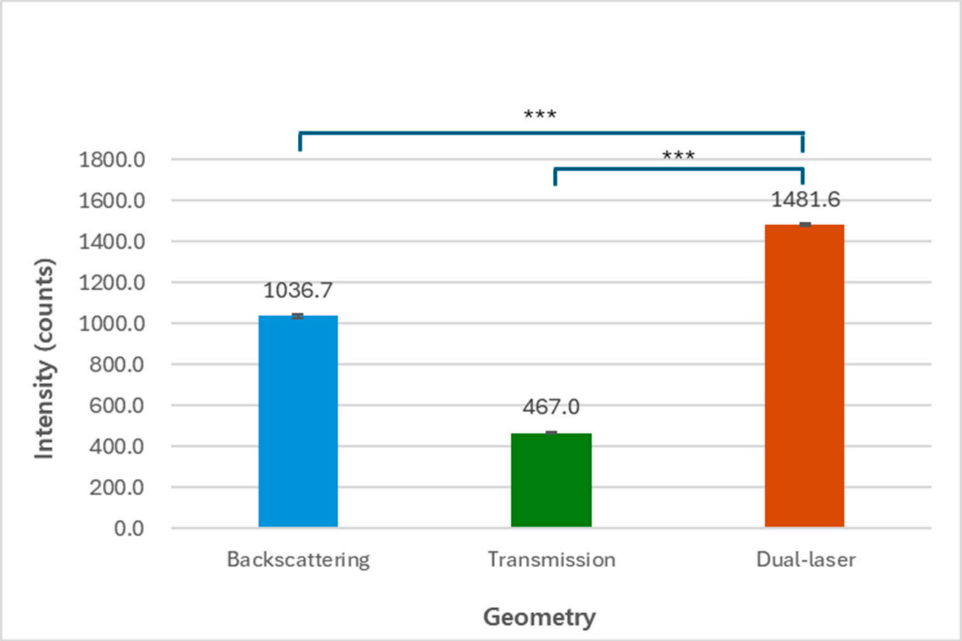

Figure S1. Comparison of Raman signal intensities for a single PS bead across different excitation geometries. The data represents the average intensities obtained under backscattering, transmission, and dual-laser configurations based on three independent measurements (n = 3) for each setup to ensure statistical reliability.

Raman intensity (counts)

	Backscattering	Transmission	Dual-laser
1	1026.6	467.8	1488.3
2	1039.3	470.1	1483.9
3	1044.3	463.2	1472.6
Average	1036.7	467.0	1481.6

***t*-test**

- Backscattering vs. Dual-laser : $p = 4.47\times10^{-7}$ ($p < 0.001$, statistically significant)
- Transmission vs. Dual-laser : $p = 9.45\times10^{-7}$ ($p < 0.001$, statistically significant)

Table S1. Raw data of Raman intensities (counts) and statistical analysis (Student's two-tailed *t*-test) evaluating the signal enhancement of the dual-laser configuration compared to conventional single-geometry setups.

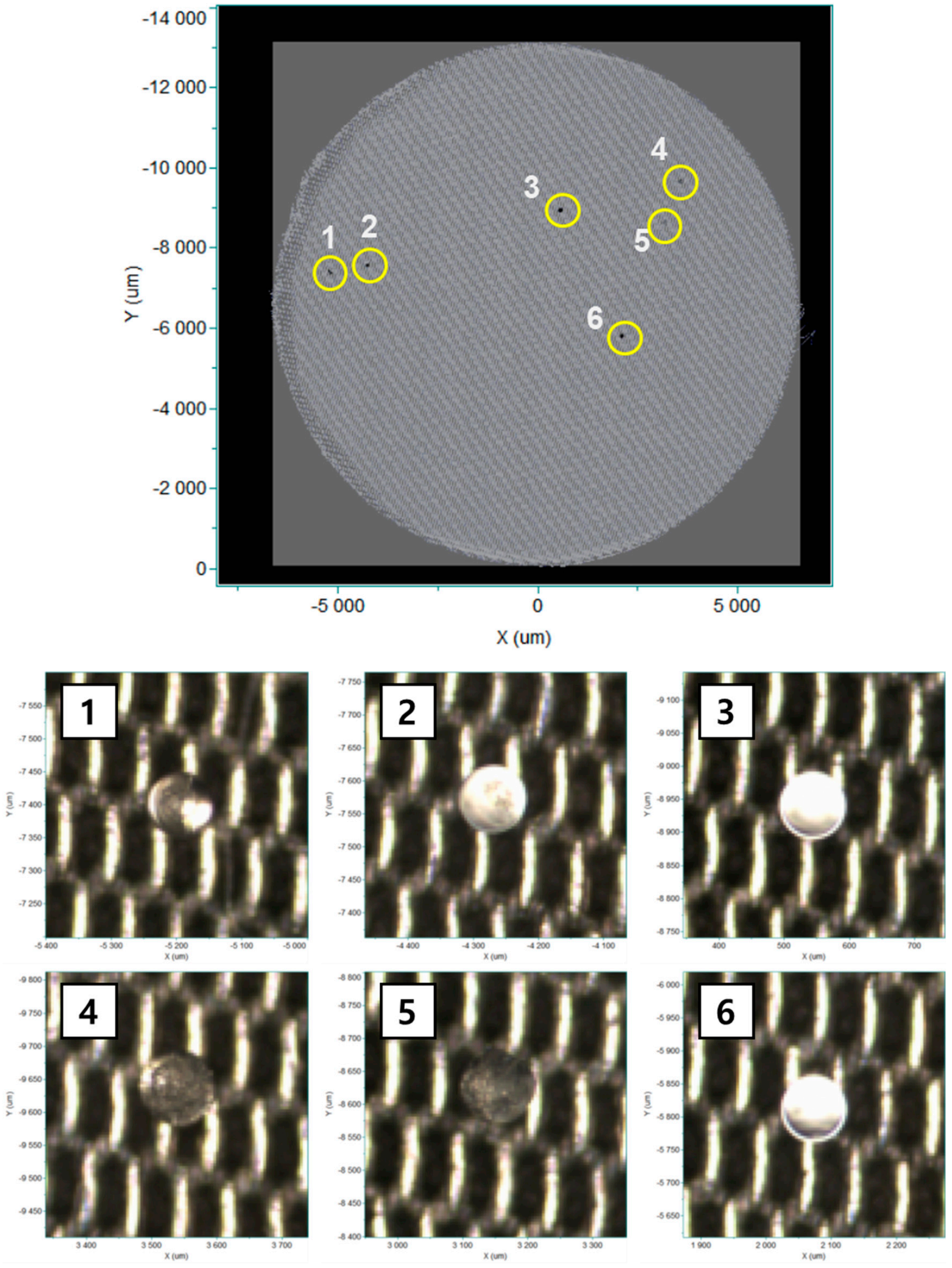

Figure S2. Optical microscope image of the successfully recovered six PS beads on a mesh filter. While transparent PS beads inherently exhibit low visual contrast against the filter background in brightfield microscopy, careful physical observation confirmed the presence of exactly six particles (highlighted in yellow circles). This physical count perfectly matches the number of real-time Raman detection events, establishing a robust ground truth for quantitative accuracy.

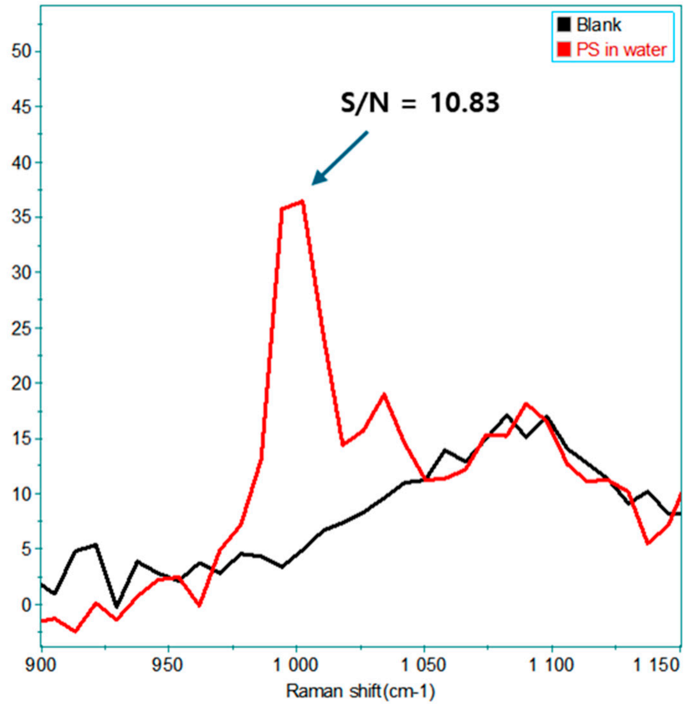

$$S/N = \frac{I_{peak} - I_{blank,mean}}{\sigma_{blank}}$$

PS peak at 1002 cm^{-1}		
I_{peak}	:	36.45 counts
$I_{blank,mean}$	:	5.79 counts
σ_{blank}	:	2.83 counts
S/N ratio		10.83

Figure S3. Visual validation of the signal-to-noise (S/N) threshold for real-time particle detection. The raw Raman spectrum of a representative PS bead (red) is overlaid with the background spectrum of ultrapure water (black). The characteristic peak at 1002 cm^{-1} demonstrates an S/N ratio of 10.83, which significantly exceeds the detection threshold of 3.
